# An alginate-layer technique for culture of *Brassica oleracea* L. protoplasts

**DOI:** 10.1007/s11627-012-9431-6

**Published:** 2012-03-30

**Authors:** Agnieszka Kiełkowska, Adela Adamus

**Affiliations:** Department of Genetics, Plant Breeding and Seed Science, University of Agriculture in Krakow, Al. 29-Listopada 54, 31-425 Krakow, Poland

**Keywords:** *Brassica alba*, *rubra*, s*abauda*, Calcium alginate, Hypocotyl, Mesophyll

## Abstract

Ten accessions belonging to the *Brassica oleracea* subspecies *alba* and *rubra*, and to *B. oleracea* var. *sabauda* were used in this study. Protoplasts were isolated from leaves and hypocotyls of *in vitro* grown plants. The influence of selected factors on the yield, viability, and mitotic activity of protoplasts immobilized in calcium alginate layers was investigated. The efficiency of protoplast isolation from hypocotyls was lower (0.7 ± 0.1 × 10^6^ ml^−1^) than for protoplasts isolated from leaf mesophyll tissue (2 ± 0.1 × 10^6^ ml^−1^). High (70–90%) viabilities of immobilized protoplasts were recorded, independent of the explant sources. The highest proportion of protoplasts undergoing divisions was noted for cv. Reball F1, both from mesophyll (29.8 ± 2.2%) and hypocotyl (17.5 ± 0.3%) tissues. Developed colonies of callus tissue were subjected to regeneration and as a result plants from six accessions were obtained.

## Introduction

The economic and agronomic importance of *Brassica* species is well known, due to their use as vegetable and oil seed crops, and as sources of condiments (Fahleson et al. 1994). Conventional breeding in *Brassica* spp. has delivered improvements of both quality and yield, but corresponding enhancements of traits such as disease resistance have been restricted by the limited availability of suitable genes in the germplasm pool, and with the problems of transferring of such traits (Radchuk et al. 2002). With the development of techniques for enzymatic isolation of protoplasts and regeneration, a valuable tool for plant genetic manipulation has become available.

Studies of protoplasts isolation in *Brassica* spp. started in the early 1980s. The majority of available reports on isolation of protoplasts and subsequent somatic fusions with protoplasts from other members of the *Brassicaceae* family, as well as distant species were focused mainly on oilseed rape (*Brassica napus*; Yarrow et al. 1990; Hansen and Earle 1994; Heath and Earle 1997; Yamagishi et al. 2002). This species is important for vegetable oil production, and genetic manipulation aimed at modification of the fatty acid composition, and has been targeted for broadening of competitiveness within the market (Wang et al. 2003).

The diploid species *Brassica oleracea* is highly morphologically variable and includes vegetable crops such as kale, cauliflower, cabbage, Brussels sprouts, and kohlrabi. Protoplast isolation and protoplast-derived plant regeneration protocols have been reported for *B. oleracea*, and somatic hybridization has been used to transfer genes for resistance to diseases such as *Alternaria brassicae* from *Sinapis alba* L. (Hansen and Earle 1997) and *Capsella bursa-pastoris* (Sigareva and Earle 1999), *Xanthomonas campestris* pv. *campestris* resistance from *B. napus* (Hansen and Earle 1995), and *Ervinia carotovora* resistance from *Brassica rapa* (Ren et al. 2000). The cytoplasmic male sterility system has been introduced to *B. oleracea* through asymmetric protoplast fusion (Sigareva and Earle 1997). Positive results, as mentioned above, have been achieved using *B. oleracea* accessions that were selected for high plant regeneration capacity, but the ability to undergo meiotic divisions and regenerate shoots remained problematic (Jourdan and Earle 1989; Zhao et al. 1995; Hansen et al. 1999a). Although intensive screening of protoplast culture conditions has been performed, the effects of genotype have still overridden most efforts to improve protocols for plant regeneration from protoplast culture. The major obstacle for commercial application of protoplast fusion in *B. oleracea* has been the lack of breeding lines with high capacity for plant regeneration from *in vitro* cultures (Holme et al. 2004).

Protoplasts derived from cell wall digestion with enzyme preparations are highly vacuolated and very fragile, hence being difficult to culture. Immobilization of protoplasts, involving entrapment of cells in a polymerizing matrix, is a relatively easy and cheap method that helps to protect the isolated cells and maintain the culture. Available data suggests that immobilization in a semisolid matrix, such as calcium alginate, allows undisturbed rebuilding of the cell wall, prevents cell agglutination, and promotes completion of mitotic divisions (Hall et al. 1993; Dirks et al. 1996; Dovzhenko et al. 1998; Pati et al. 2005). To our knowledge, immobilization of protoplasts in alginate layers has never been utilized for *B. oleracea*, and only one report of its use has been made for *B. napus*, with division frequencies in the 3–5% range (Dovzhenko 2001).

The objective of this study was to analyze yields, viability, and division frequencies of *Brassica oleracea* protoplasts embedded in calcium alginate, and to establish *in vitro* culture conditions for effective shoot regeneration.

## Materials and Methods

### Plant material.

The following sources of mesophyll protoplasts were used: *B. oleracea* var. *capitata f. alba* cultivars Kamienna Głowa (PlantiCo, Poland), Amager (PNOS, Ożarów Mazowiecki, Poland), Kilaxy F1 (Syngenta Seeds), Benia F1 (POLAN, Poland); *B. oleracea* var. *capitata f. rubra* cv. Reball F1 (Syngenta Seeds), Rodeo F1 (Nickerson Zwaan, Netherlands); *B. oleracea* var*. sabauda* (Savoy cabbage) cv. Vertus F1 (PlantiCo). Seeds of all mentioned cultivars were commercial samples. Mesophyll protoplasts were also isolated from three DH lines (No. 582, 1071, 5071) obtained via microspore embryogenesis from the *B. oleracea* var. *capitata f. rubra* in the Dept. of Genetics, Plant Breeding and Seed Science in Krakow, Poland. Hypocotyl protoplasts were isolated from cultivars Kamienna Głowa, Kilaxy F1, and Reball F1.

Seeds of all accessions were sterilized in 70% (*v/v*) ethanol for 2–3 min, 10% (*w/v*) chloramine T (Biochemie Poland, Katowice, Poland) for 20 min, and washed three times with sterile distilled water for 5 min. Seeds were germinated on MS (Murashige and Skoog 1962) medium supplemented with 0.8% (*w/v*) agar (Biocorp, Warszawa, Poland). In order to obtain plantlets with well-developed leaves, seeds were placed in sterile 250 ml glass jars containing 30 ml of MS medium, and kept at 26 ± 2°C under 16 h photoperiod with light intensity 55 μmol m^−2^ s^−1^. Etiolated hypocotyls were obtained from seeds placed in 9 cm Petri dish with 5 ml of MS medium, kept in the dark at 26 ± 2°C.

### Protoplast isolation and culture.

Newly expanded young leaves of 2-, 4-, and 6-wk-old plants, and hypocotyls from 1- to 2-wk-old seedlings were used. In both cases, 1 g of plant tissue was placed on a glass Petri dish in the presence of 0.5 M mannitol at pH 5.8 (plasmolysis solution), was cut into fine pieces and incubated for 1 h in the dark at 25°C. The suspension was then treated with the selected enzyme solution and incubated for 16–18 h in the dark at 25°C with gentle shaking (30 rpm). For digestion of protoplasts from mesophyll and hypocotyls of tested cultivars, ES enzyme solution consisting of KM (Kao and Michayluk 1975) medium, 0.1% (*w/v*) macerozyme R-10 (Duchefa Biochemie, Netherlands), 1.0% (*w/v*) cellulase Onozuka R-10 (Duchefa Biochemie), and 0.8 M sucrose pH 5.6 was used. For protoplast isolation from mesophyll of the DH lines, beside ES enzyme solution, ES IV and D solutions were used. The ES IV enzyme solution consisted of 1.0% (*w/v*) cellulase Onozuka R-10, 0.1% (*w/v*) pectolyase Y-23 (Duchefa Biochemie, NL), 0.6 M mannitol, 20 mM 2-(*N*-morpholino)ethanesulfonic acid (MES; Sigma), 5.0 mM MgCl_2_·6 H_2_0, pH 5.6; D solution consisted of 1.0% (*w/v*), cellulase Onozuka R-10, 0.5% (*w/v*) Driselase® (Duchefa Biochemie), 1.0% (*w/v*) macerozyme R-10, 0.6 M mannitol, 20 mM MES, 5.0 mM MgCl_2_·6H_2_0, pH 5.6. All enzyme solutions were filter-sterilized (0.22 μm, Millipore, Durham, UK).

The isolated protoplasts were filtered through a 100 μm nylon mesh (Millipore, Durham, UK) and centrifuged at 1,000 rpm for 5 min. Pellets were resuspended in 0.5 M sucrose with 1 mM MES, overlaid with 2 ml of W5 salt solution (Menczel et al. 1981), and centrifuged for 10 min at 1,200 rpm. Protoplasts, now localized in the interphase between two solutions, were collected into a new tube with addition of W5 solution, and centrifuged at 1,000 rpm for 5 min. The protoplast pellet was then resuspended in culture medium, and protoplast yield was counted using a hemocytometer. Density of cultured protoplasts was adjusted to 8 × 10^5^ per milliliter of culture medium. The protocol of Damm and Willmitzer (1988) with modification of Grzebelus et al. (2012) for protoplast immobilization in a calcium alginate layer was employed. A suspension of protoplast and alginate solution consisting of 2.8% (*w/v*) Na-alginate and 0.4 M mannitol was prepared by gentle mixing in equal volumes to obtain a final density of protoplasts in the culture of 4 × 10^5^ ml^−1^. Approximately 0.5 ml of protoplast–alginate mixture was gently spread onto 1% (*w/v*) agar containing 20 mM CaCl_2_. After 1 h incubation at room temperature, alginate disks with immobilized protoplasts were formed. The disks were transferred to the 6 cm Petri dish containing 4 ml of culture medium.

Two types of culture medium were used: CPP medium according to Dirks et al. (1996) supplemented with 0.1 mg l^−1^ 2,4-dichlorophenoxyacetic acid (2,4-D) and 0.2 mg l^−1^ zeatin, pH 5.6; and 8P medium according to Glimelius (1984) supplemented with 1.0 mg l^−1^ 2,4-D, 0.1 mg l^−1^ 1-naphthaleneacetic acid, and 0.5 mg l^−1^ benzyladenine, pH 5.6. Both media were filter sterilized (0.22 μm; Millipore, Durham, UK). The cultivars were cultured on CPP medium exclusively, whereas DH lines on CPP and 8P medium. Culture media were renewed after 10 d. Cultures were incubated in the dark at 26 ± 2°C.

Protoplast viability was estimated on the day of isolation (0 h), and at 24 and 48 h of culture, by staining with fluorescein diacetate according to Anthony et al. (1999). Metabolically active protoplasts were visualized by excitement of the accumulated fluorescein under blue light excitation at 485 nm with a 515 nm barrier filter, and observations were made using Axiovert S 100 (Carl Zeiss, Göttingen, Germany). Viable protoplasts exhibited yellow–green fluorescence, while nonviable protoplasts were invisible.

### Plant regeneration.

Colonies of protoplast-derived calli were freed from alginate layers according to the protocol of Damm and Willmitzer (1988). Callus colonies were transferred separately from each layer to 250 ml jars containing regeneration media, of which three were used: MS medium consisting of MS micro- and macro-elements and vitamins with 20 g l^−1^ sucrose, free of plant growth regulators (PGRs); R1—consisting of MS micro- and macro-elements and vitamins with 0.4 mg l^−1^ calcium panthothenate, 0.1 mg l^−1^ gibberelic acid (GA_3_)_,_ 3.0 mg l^−1^ kinetin, and 30 g l^−1^ sucrose; R2—consisting of MS micro- and macro-elements, and modified vitamin composition; 0.5 mg l^−1^ nicotinic acid, 0.1 mg l^−1^ pyridoxine and thiamine, 3.0 mg l^−1^ glycine, and 20 g l^−1^ sucrose. The media were adjusted to a pH of 5.7–5.8 and 0.25% (*w/v*) Phytagel was added (Sigma) prior to autoclaving (20 min at 121°C; 1.4 × 10^4^ kg m^−2^). Developing shoots were transferred to fresh medium every 3–4 wk. Cultures were maintained at 26 ± 2°C with 16 h photoperiod at a light intensity of 55 μmol m^−2^ s^−1^.

### Statistical analysis.

A single treatment consisted of five Petri dishes in three replicates. Each experiment was replicated three times. Protoplast yield was expressed as the number of protoplasts per gram of fresh weight (fw) of initial tissue. Viability of protoplasts was scored as number of protoplasts with green fluorescence per total number of observed cells (×100). The means were calculated on the basis of a minimum of 200 cells per Petri dish. Division frequency of alginate-embedded protoplasts was expressed as the number of dividing protoplasts per total number of observed protoplasts (×100), counted on days 5, 10, and 15 of culture. The regeneration frequency was calculated as the number of shoots regenerated from callus per total number of calli cultured on the regeneration medium (×100). Analyses were performed using Statistica ver. 9.0 (Statsoft Poland Inc., Poland), using multivariate analysis of variance module. Mean separation was performed using Tukey’s honestly significant difference.

## Results

### Yield of protoplasts.

Protoplasts were successfully isolated from mesophyll tissue from all tested accessions. Protoplasts from white forms and Savoy cabbage were spherical and rich in chloroplasts that were randomly distributed in the cytosol. Protoplasts isolated from red forms (cv’s. Rodeo F1, Reball F1, and DH lines) had bright green or purple interiors due to the presence of anthocyanins in the cell vacuoles. The yield of leaf protoplasts after isolation varied between tested accessions and was on average 2.0 ± 0.1 × 10^6^ per gram of fresh tissue (Table 1). The highest yields of leaf protoplast were noted for red forms of cabbage cv. Reball F1 and Rodeo F1 (being 3.2 ± 0.2 × 10^6^ and 2.6 ± 0.4 × 10^6^, respectively). Higher yields of protoplasts were obtained from leaves of 4- and 6-wk-old *in vitro* grown plants.

**Table 1.** Mean effect of accession, age of explant, and enzyme solution on the yield of protoplasts (×10^6^ g^−1^ fw) isolated from leaf mesophyll and hypocotyls of cultivars and DH lines of cabbage (±SE)

Factor	Source of protoplasts	
	Leaves	Hypocotyls
Accession		
Kamienna Głowa	2.1 ± 0.3 b, c, d	0.7 ± 0.1 a, b
Amager	1.4 ± 0.3 c, d	n/a
Kilaxy F1	1.7 ± 0.3 b, c, d	0.6 ± 0.1 b
Benia F1	1.8 ± 0.4 b, c, d	n/a
Rodeo F1	2.6 ± 0.4 a, b	n/a
Reball F1	3.2 ± 0.2 a	0.8 ± 0.1 a
Vertus F1	2.3 ± 0.4 b, c	n/a
Line 582	1.9 ± 0.2 b, c, d	n/a
Line 1071	1.3 ± 0.2 d	n/a
Line 5071	2.0 ± 0.1 b, c, d	n/a
Age of explant		
1 Week old	n/a	0.7 ± 0.1 a
2 Weeks old	1.8 ± 0.2 b	0.7 ± 0.1 a
4 Weeks old	2.1 ± 0.1 a	n/a
6 Weeks old	2.0 ± 0.2 a	n/a
Mean	2.0 ± 0.1	0.7 ± 0.1
Enzyme solution^a^		
D	1.4 ± 0.2 b	n/a
ES	2.1 ± 0.1 a	n/a
ESIV	1.8 ± 0.1 a, b	n/a

Values in each *column* followed by the same *letter* are not significantly different (*p* ≤ 0.05, Tukey’s honestly significant difference)

^a^Applied only to DH lines, therefore excluded below mean

Mesophyll protoplasts were isolated from three doubled haploid lines using the same protocol as formerly described for cultivars, and in addition, three enzyme solutions and two culture media were used. Measurement of protoplasts was not performed in this study. However, based on the microscopic observations, protoplasts isolated from DH lines were smaller in comparison to mesophyll protoplasts isolated from tested cultivars. The yield of protoplasts from 1 g of fresh tissue of lines 582 and 5071 was similar to that obtained for cultivars Kamienna Głowa, Kilaxy F1, and Benia F1, and was on average 2.0 × 10^6^ (Table 1). The lowest yield, at an average of 1.3 ± 0.2 × 10^6^ g fw^−1^, was obtained for line 1071. The enzyme solution influenced the yield of protoplasts of tested DH lines. A larger number of protoplasts (2.1 ± 0.1 × 10^6^ g fw^−1^) were isolated in the presence of ES enzyme solution. The lowest yield of protoplasts (1.4 ± 0.2 × 10^6^ g fw^−1^) was obtained when digestion was performed using mixture containing 0.5% (*w/v*) Driselase®.

The hypocotyl-derived protoplasts were isolated from three selected *B. oleracea* cultivars, representing both white and red forms. The average yield of hypocotyl protoplasts was almost threefold lower (0.7 ± 0.1 × 10^6^ g fw^−1^) than from mesophyll sources isolated from the same cultivars (Table 1). Differences in the yield of protoplasts were observed between tested cultivars. The highest yield (0.8 ± 0.1 × 10^6^ g fw^−1^) was obtained from red cabbage cv. Reball F1. No influence of the age of explant on the yield of hypocotyl-derived protoplasts was observed.

### Viability of cabbage protoplasts immobilized in calcium alginate.

**Figure 1.**
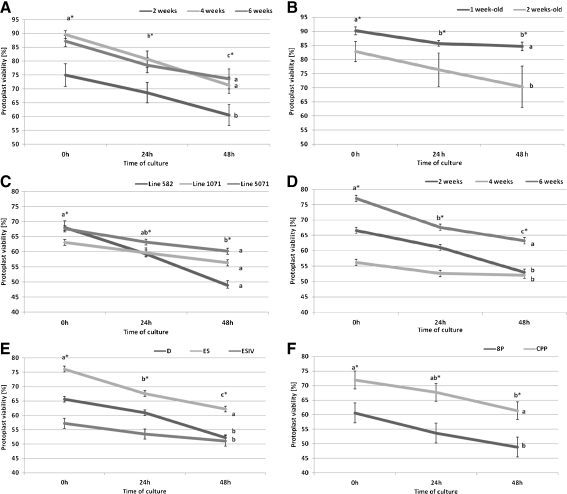
Protoplast viability. The effect of the age of the explant on viability of protoplasts isolated from leaf mesophyll (*A*) and hypocotyls (*B*) of tested cabbage cultivars (±SE). The effect of the genotype (*C*), age of explant (*D*), enzyme solution (*E*), and culture medium (*F*) on the viability of protoplast isolated from leaf mesophyll of DH lines of cabbage (±SE). Values followed by the same *letter* are not significantly different (*p* ≤ 0.05, HSD). *Letters* with *asterisks* indicate significant differences in dependency on time of culture, remaining tested factor.

On the day of isolation, viability of the leaf and hypocotyl protoplasts embedded in alginate was high, at an average of 85–90% for the cultivars (Fig. 1*A*, *B*) and 60–80% for the DH lines (Fig. 1*C–F*). The next day, however, a decrease of viability to about 5–8% was observed. There were no differences in viability of leaf-derived protoplast between tested accessions, but we observed differences determined by explant age. The highest viability, reaching about 90% on the day of isolation, was recorded for 4- and 6-wk-old mesophyll (Fig. 1*A*)-derived protoplasts and 1-wk-old hypocotyl (Fig. 1*B*)-derived protoplasts. The highest percent of viable protoplasts was noted from 6-wk-old DH line plantlets (Fig. 1*D*), when digestion was performed using ES enzyme solution (Fig. 1*E*) and when protoplasts were cultured on CPP medium (Fig. 1*F*).

### Division frequency of cabbage protoplasts immobilized in calcium alginate.

Around the third and fourth day of culture, protoplasts started to change shape and the first mitotic divisions were observed. On the fifth day, an average of 3.7 ± 0.4% cells isolated from leaves underwent the first division (Table 2). The division frequency increased with the duration of culture, and on the 15th day, reached an average of 9.9 ± 1.0%. Differences in ability to undergo divisions were identified, dependent on the accession. The highest percent (29.8 ± 2.2%) of dividing protoplasts was scored for cv. Reball F1 on day 15 of culture. Divisions in the range of 14–16% were observed for white form cultivars Kilaxy F1 and Kamienna Głowa. The mitotic activity of protoplasts isolated from DH lines was relatively low and varied from 1.1 ± 0.5% (line 582) to 5.8 ± 1.3% (line 5071), on the 15th day of culture. There were no differences in division frequency of protoplasts isolated from 4- and 6-wk-old plantlets, and only 5.6 ± 1.8% of protoplasts isolated from younger tissues underwent divisions after 2 wk of culture. The highest percent (4.9 ± 2.0%) of dividing protoplasts of tested DH lines was observed on CPP medium.

**Table 2.** Average effect of the accession, age of explants and culture medium on division frequency (%) of protoplasts isolated from leaf mesophyll of cultivars and DH lines of cabbage (±SE)

Factor	Time of culture		
	5 Days	10 Days	15 Days
Accession			
Kamienna Głowa	6.5 ± 0.9 a	10.2 ± 1.1 a, b	14.5 ± 1.7 b
Amager	4.0 ± 1.5 a, b, c	5.7 ± 2.2 b, c, d	8.3 ± 2.7 b, c, d
Kilaxy F1	4.9 ± 0.6 a, b	11.9 ± 2.4 a, b	16.8 ± 4.0 b
Benia F1	3.5 ± 1.0 a, b, c	6.3 ± 1.9 b, c, d	9.2 ± 2.5 b, c, d
Rodeo F1	6.2 ± 1.5 a, b	9.3 ± 2.3 a, b, c	13.3 ± 3.5 b, c
Reball F1	7.8 ± 2.0 a	15.3 ± 2.8 a	29.8 ± 2.2 a
Vertus F1	3.3 ± 1.6 a, b, c	5.3 ± 2.1 b, c, d	7.0 ± 2.6 b, c, d
Line 582	0.2 ± 0.1 c	0.5 ± 0.2 d	1.1 ± 0.5 d
Line 1071	1.2 ± 0.4 b, c	1.8 ± 0.5 c, d	2.8 ± 0.6 c, d
Line 5071	2.1 ± 0.5 b, c	3.8 ± 0.8 c, d	5.8 ± 1.3 c, d
Age of explant			
2 Weeks old	1.5 ± 0.3 b	3.2 ± 1.0 b	5.6 ± 1.8 b
4 Weeks old	4.6 ± 0.6 a	7.6 ± 0.9 a	11.7 ± 1.5 a
6 Weeks old	4.3 ± 0.7 a	8.0 ± 1.3 a	11.2 ± 2.0 a
Mean	3.7 ± 0.4 a	6.5 ± 0.7 b	9.9 ± 1.0 c
Culture medium^a^			
8P	0.6 ± 0.4 b	1.1 ± 0.7 b	1.6 ± 0.8 b
CPP	1.7 ± 0.9 a	3.1 ± 1.3 a	4.9 ± 2.0 a

Values in each *column* followed by the same *letter* are not significantly different (*p* ≤ 0.05, Tukey’s honestly significant difference)

^a^Applied only to DH lines, therefore excluded below mean

The division frequency of hypocotyl-derived protoplasts depended on the cultivar and the age of explants (Table 3), and interactions between these factors were observed. The highest percent (17.5 ± 0.3%) of dividing protoplasts was identified from 2-wk-old hypocotyls of cv. Reball F1, while for cv. Kamienna Głowa, superior results (9.0 ± 1.4%) were obtained from 1-wk-old explants.

**Table 3.** Interaction of cultivar and age of explants on the division frequency (%) of protoplast isolated from hypocotyls of selected cultivars of cabbage (±SE)

Explant age	Cultivar	Time of culture		
		5 Days	10 Days	15 Days
1 Week old	Kamienna Głowa	2.5 ± 0.7 a	4.5 ± 0.7 b	9.0 ± 1.4 b
	Kilaxy F1	4.5 ± 0.5 a	6.0 ± 1.0 b	7.5 ± 2.0 b, c
	Reball F1	2.5 ± 0.7 a	5.5 ± 0.2 b	8.0 ± 1.4 b, c
2 Weeks old	Kamienna Głowa	3.0 ± 0.8 a	4.0 ±2.1 b	5.0 ± 2.1 b, c
	Kilaxy F1	1.5 ± 0.4 a	3.0 ± 0.0 b	3.0 ± 0.0 c
	Reball F1	6.0 ± 0.7 a	10.5 ± 1.4 a	17.5 ± 0.3 a
Mean		3.3 ± 0.6 c	5.6 ± 0.9 b	8.3 ± 1.2 a

Values in each *column* followed by the same *letter* are not significantly different (*p* ≤ 0.05, Tukey’s honestly significant difference)

Multiple cell colonies started to become visible to the naked eye at around the fourth wk of culture. Differences in the size of colonies were observed, caused by lack of synchrony in the mitotic divisions, consequently impacting colony development.

### Plant regeneration.

Colonies of calli were transferred to solid regeneration media. After approximately 4 wk, colonies started to differentiate, and formation of small green or purple-green shoots was observed. The regeneration ability was accession-dependent, and varied from 0.0 to 34.6 ± 4.9% (Table 4). Colonies of calli from tested DH lines failed to regenerate. Calli tissue of cv. Amager only produced roots after transfer to the regeneration media, and no shoot regeneration was observed irrespective of the medium composition. The highest frequency of callus differentiation (29.9 ± 5.0%) was recorded on the MS medium in the absence of PGRs. On this media, plantlets from six tested accessions (Kamienna Głowa, Kilaxy F1, Benia F1, Reball F1, Rodeo F1, and Savoy cabbage cv. Vertus F1) were regenerated. Shoot regeneration of cultivars Kamienna Głowa, Reball F1, and Vertus F1 was recorded on the R1 medium, while on the R2 medium, only calli from cultivars Kamienna Głowa and Reball F1 were converted into plantlets. Differences in regeneration were dependent on the source of protoplasts. A higher percent (26.2 ± 4.2%) of regenerated shoots was observed from hypocotyl-derived protoplasts.

**Table 4.** Effect of source tissue, accession, and media on the efficiency (%) of shoot regeneration from the protoplast-derived callus colonies of different cabbage accessions (±SE)

Factor	Source of protoplasts		
	Leaves	Hyopcotyls	Mean
Accession			
Kamienna Głowa	36.8 ± 5.8 a, b	22.8 ± 5.1 a	29.8 ± 5.5 a, b
Amager	0.0 d	n/a	0.0 e
Kilaxy F1	24.5 ± 3.5 a, b, c	28.1 ± 4.2 a	26.4 ± 3.9 a, b, c
Benia F1	16.4 ± 1.9 c, d	n/a	16.4 ± 1.9 c, d
Rodeo F1	11.4 ± 1.6 c, d	n/a	11.4 ± 1.6 d
Reball F1	41.6 ± 6.5 a	27.6 ± 3.3 a	34.6 ± 4.9 a
Vertus F1	22.2 ± 3.4 b, c	n/a	22.2 ± 3.4 b, c, d
Line 582	0.0 d	n/a	0.0 e
Line 1071	0.0 d	n/a	0.0 e
Line 5071	0.0 d	n/a	0.0 e
Regeneration medium			
MS	19.8 ± 5.1 a	40.0 ± 4.8 a	29.9 ± 5.0 a
R1	15.6 ± 3.6 a, b	25.7 ± 3.0 b	20.7 ± 3.3 b
R2	10.6 ± 2.7 b	13.0 ± 4.7 c	11.8 ± 3.7 c
Mean	15.3 ± 3.8 b	26.2 ± 4.2 a	

Values in each *column* followed by the same *letter* are not significantly different (*p* ≤ 0.05, Tukey’s honestly significant difference)

## Discussion

Protoplasts from *Brassica* species were isolated from different sources, such as cell suspensions (Simmonds et al. 1991), leaves (Jourdan and Earle 1989), roots (Xu et al. 1982), petioles (Bornman 1985), cotyledons (Lu et al. 1982; Zhao et al. 1995), hypocotyls (Chuong et al. 1985), stem embryos (Kohlenbach et al. 1982), stem peels (Chuong et al. 1987), and stem cortex (Klimaszewska and Keller 1987). The use of some types of tissues (such as roots and cortex) leads to a higher risk of contamination, while others (such as hypocotyls and cotyledons) required the use of large amount of seeds to obtain satisfactory yields, and others (such as suspension cultures) are difficult to maintain for *Brassica* species (Simmonds et al. 1991). In *B. oleracea*, protoplasts have previously been isolated mainly from leaves (Fu et al. 1985; Robertson and Earle 1986; Kaur et al. 2006) and hypocotyls (Glimelius 1984; Mukhopadhyay et al. 1991; Chen et al. 2004). In this study, both of these sources, taken from sterile conditions, were utilized and protoplasts were readily isolated for all tested accessions. The efficiency of isolation obtained was relatively high. The number of protoplasts released from leaf tissues was similar to the value reported for other *Brassica* species, for which yield was generally in the range of 1–4 × 10^6^ per gram of fw (Robertson and Earle 1986; Davey et al. 2005b). The mean yield of hypocotyl-derived protoplasts reported for cabbage is on average 1–3.5 × 10^5^ per gram of tissue (Glimelius 1984; Chen et al. 2004). Using the present protocol, higher yields (0.7 ± 0.1 × 10^6^) of protoplasts were obtained from the same amount of tissue.

The DH lines of cabbage used in this study exhibited lower seed vigor and differing performance (smaller and more compact leaf, less roots) than for tested cultivars. The yield as well as protoplast viability of tested DH lines was also reduced in comparison to the cultivars. Enzymatic solutions containing cellulase, pectolyase, macerozyme, and Driselase® were used in this experiment. The first three enzymes are typically used for tissue digestion in *Brassica* sp. protoplast isolation protocols, but Driselase® has been used sporadically. Robertson and Earle (1986) reported that use of an enzymatic solution containing 0.5% (*w/v*) Driselase® for broccoli protoplasts isolation increased the yield, but this observation was not confirmed in our study. Protoplasts from DH lines were cultured on two media. The frequencies of divisions obtained on CPP medium were higher than those on 8P, possibly due to the presence of lower (0.1 mg l^**−**1^) concentration of 2,4-D. The beneficial influence of different levels of 2,4-D in the culture medium was reported for *Brassica* sp. (Kohlenbach et al. 1982; Glimelius 1984). However, our results suggest that lower concentrations were more beneficial for stimulation of cell divisions for tested DH lines. Relatively good yields of viable protoplasts were obtained, but despite testing various culture conditions, it was difficult to obtain protoplasts which underwent divisions at high frequencies from tested DH lines.

Young tissues from *in vitro* conditions generally obtained optimal results from *Brassica* sp. However, successful protoplast culture and plantlet regeneration from greenhouse-grown plants has been also reported (Shillito et al. 1983; Fu et al. 1985). In general, to obtain optimal amount and quality of tissue from *in vitro* sown seeds, a period of 1 mo is required. Such plantlets are young, vigorous and healthy, and therefore the best source of material for culture. In the present study, the highest yield of viable protoplasts undergoing divisions was obtained from the leaf tissue of 4- to 6-wk-old plantlets. Higher proportions of viable hypocotyl-derived protoplasts were observed from 1-wk-old explants, but the division frequency of such protoplasts was both age- and accession-dependent. The use of younger hypocotyls was also reported, but sowing of more seeds is required to obtain sufficient yields (Glimelius 1984; Mukhopadhyay et al. 1991; Chen et al. 2004), and division frequency may be lowered (Fu et al. 1985; Robertson et al. 1988).

Some studies suggest that with higher culture densities, higher plating efficiencies could be achieved (Chuong et al. 1985; Vamling and Glimelius 1990). Cultured protoplasts stimulate mitotic division of adjacent cells by releasing growth factors, including amino acids, into the surrounding medium (Davey et al. 2005a). The optimum plating density for *B. oleracea* is usually 5 × 10^4^ protoplasts per milliliter of culture medium (Pelletier et al. 1983; Glimelius 1984; Robertson and Earle 1986). In this study, a higher culture density (4 × 10^5^ ml^**−**1^) was applied. The high density of the culture may be responsible for decreasing viability of protoplasts as observed in our study. Considering the frequencies of divisions that were obtained, it seems likely that denser cultures rapidly depleted available nutrients and a large number of protoplasts failed to undergo divisions.

The timing of the first mitotic division is generally expected between 24 and 48 h of culture (Glimelius 1984; Mukhopadhyay et al. 1991), but a shift of the first mitosis to days 3 or 4 of culture, as observed in our study, was also reported for *B. oleracea* (Fu et al. 1985; Robertson and Earle 1986; Kaur et al. 2006). Division frequencies in *B. oleracea* varied as a consequence of the source of tissue, culture system, culture maintenance, and genotype. The average division frequency reported for *Brassica* spp. in the vicinity of second week of culture varied from 0% to 40% (Glimelius 1984; Zhao et al. 1995; Chen et al. 2004). Colony formation is usually established after 20–30 d. The size of colonies at the time of transfer is important and not dependent on the protoplast source. Callus colonies that are too small (<2 mm) generally do not differentiate, and eventually become brown and die. In contrast, larger colonies are able to form shoot primordia, and regeneration of plants from such colonies is much more effective (Glimelius 1984; Fu et al. 1985; Kaur et al. 2006).

*Brassica* protoplast cultures are usually maintained in liquid media, requiring three to four medium replacements during the culture period (Pelletier et al. 1983; Glimelius 1984; Robertson and Earle 1986; Mukhopadhyay et al. 1991). Studies revealed that in the liquid culture, protoplasts tend to aggregate and release brown particles (phenolic compounds) into the medium; moreover, the aggregates tend to fall to the bottom of the Petri dish, consequently decreasing protoplast divisions and causing death (Klimaszewska and Keller 1987; Mukhopadhyay et al. 1991; Kaur et al. 2006). To prevent aggregation, browning, and necrosis in the culture, and to improve plating efficiency, various protoplast culture systems were applied. The use of agarose (Yamashita and Shimamoto 1989), combining liquid and solidified (agarose) phases (Klimaszewska and Keller 1987), as well as use of feeder layers (Walters and Earle 1990) and nurse cultures (Chen et al. 2001) has been reported. Agarose has assisted prevention of aggregation and necrosis of *B. juncea* protoplasts, but has no beneficial effect on protoplasts of *B. oleracea* (Chen et al. 2004). Immobilization in alginate was used for species such as *Arabidopsis thaliana*, for which frequency of divisions ranged from 0.6% to 30% (Damm and Willmitzer 1988; Dovzhenko et al. 2003), sugar beet (20–30%; Hall et al. 1993), carrot (4–70%; Dirks et al. 1996; Grzebelus et al. 2012), and tobacco (96%; Dovzhenko et al. 1998). Our study suggests that the use of alginate layers has a positive effect on cultured protoplasts, with mitotic divisions being noted for all tested accessions. However, a strong effect of genotype was observed for each of the important steps of the experiment (mitotic divisions, shoot regeneration), which determined the final results. In *B. oleracea*, many authors have emphasized the importance of genetic control in the ability to regenerate from protoplast-derived microcalli.

A genetic study indicated that the regeneration ability is controlled by two or three genes (Hansen et al. 1999b). Further studies verified the general hypothesis, reducing the number of controlling genes to two, and eliminating major dominance effects (Holme et al. 2004). Such results demonstrate that success in protoplast cultures depends on possession of genotypic constitutions with major high-response genes in available materials or introgression of these genes to recalcitrant lines (Armstrong et al. 1992; Koorneef et al. 1993; Holme et al. 2004). Average regeneration frequencies reported for *Brassica* spp. are between 0% and 55% from mesophyll protoplasts and 0–30% from hypocotyls (Glimelius 1984; Jourdan and Earle 1989; Yamashita and Shimamoto 1989; Hu et al. 1990; Ren et al. 2000; Kirti et al. 2001), but higher frequencies (80–90%) have been also reported (Mukhopadhyay et al. 1991; Delpierre and Boccon-Gibod 1992). In the present study, all regenerated plants were normal, well-developed and healthy, without variegation. The highest regeneration frequency was obtained on the MS hormone-free medium. Addition of GA_3_ and kinetin (R1) as well as modification of MS medium vitamin composition (R2) did not significantly enhance regeneration. The observed higher regeneration frequency of hypocotyl-derived protoplasts may be explained by exclusive use of the best-responding genotypes, while leaf protoplasts were isolated from wide range of genotypes with different responses to the applied culture conditions.

## Conclusions

Differentiation in the *in vitro* cultures is generally known to be genotype-specific, and *Brassica* species are no exception (Jourdan and Earle 1989; Loudon et al. 1989; Zhao et al. 1995; Hansen et al. 1999a), as confirmed in this study. Mesophyll- and hypocotyl-derived protoplasts were successfully isolated with satisfactory yields, viability, and division frequencies, especially for the red form of cabbage cv. Reball F1 and white form of cabbage cv. Kamienna Głowa, enabling use in further research. The modification of culture maintenance such as immobilization of protoplasts in extra thin alginate films (Pati et al. 2005) may be beneficial, and will be performed in the future. Further efforts to transfer resistance genes by protoplast fusion will be continued in our laboratory.
